# The Relationship between Personality and Depression in Expectant Parents

**DOI:** 10.1155/2011/356428

**Published:** 2011-08-11

**Authors:** Elda Andriola, Michela Di Trani, Annarita Grimaldi, Renato Donfrancesco

**Affiliations:** ^1^Developmental Unit, Beck Institute, Via Gioberti 54, 00185 Rome, Italy; ^2^Department of Clinical and Dynamic Psychology, Sapienza University, via dei Marsi 78, 00185 Rome, Italy; ^3^Department of Obstetrics and Gynecology, Città di Roma Hospital, via Maidalchini 20, 00152 Rome, Italy; ^4^Sandro Pertini Hospital, Asl RM B, via dei Monti Tiburtini 385, 00157 Rome, Italy

## Abstract

Several studies assessed the relationship between depression and dimensions of temperament/character using the Cloninger's model of personality and the TCI-R. The aim of this study is clarify the relation between depression and personality in men and women who are expecting a baby. The Temperament and Character Inventory—Revised Form and the Beck Depression Inventory were administered to 65 pregnant women and 37 husbands during the last quarter of pregnancy. ANOVAs showed that pregnant women had higher levels of depression, reward dependence, and self-transcendence than the expectant fathers. Hierarchical Multiple Regression Analysis in the pregnant women group showed that harm avoidance and self-directedness were significant predictors of the level of depression. In the expectant fathers, only self-directedness was a significant predictor of depression. Low TCI-R self-directedness is a strong predictor of depression in expectant parents during pregnancy regardless of gender, and high TCI-R harm avoidance is an additional predictor of depression in expectant mothers.

## 1. Introduction

The relationship between personality and mood disorders must be considered from multiple points of view because of the complexity of the development of both depression and personality. On one hand, personality features may be an antecedent influence on depression. There is evidence that personality influences the risk of depression as a predisposition or as an early attenuated expression of later disorder [1, 2]. On the other hand, current mood state can influence how people describe their personality [3].

Cloninger et al. [4, 5] developed a psychobiological model in which personality is comprised of both temperament and character traits. There are four dimensions of temperament in Cloninger's model: Harm Avoidance (HA) involves the inhibition of behavior by anxiety-provoking stimuli; Novelty Seeking (NS) involves the activation of behavior by desire to explore novelty or complexity, as well as excitability by frustration and boredom; Reward Dependence (RD) involves need for social approval and attachment; Persistence (P) is related to perseverance in behavior despite frustration and fatigue. Hence temperament involves individual differences in basic emotional impulses, such as fear (related to high HA), anger (related to high NS), disgust (related to high RD), and ambition (related to high P). There are advantages and disadvantages to both high and low extremes of temperament, and conflicts in motivation may arise when the same situation, such as expecting a baby, presents both potential difficulties and potential rewards. Consequently, the basic emotional drives of temperament are regulated by three character traits in Cloninger's model of personality development. Self-directedness (SD) is a persons' ability to self-regulate their behavior in accordance with chosen goal and values, so that they are responsible, purposeful, and resourceful. Cooperativeness (C) is the ability to get along with other people by being tolerant, emphatic, helpful, and forgiving. Self-Transcendence (ST) is a person ability to identify with nature and the world as a whole, so that a person seeks to understand what is beyond their individual human existence and is able to sublimate and act altruistically. Strong development of each of the three character dimensions has been shown to promote health and happiness generally [6]. Cloninger's Temperament and Character Inventory—Revised Form (TCI-R) is one widely used self-report measure of personality and it assessed the temperament and the character according to the Cloninger's model [7]. 

Several studies have assessed the relationship between dimensions of temperament/character and depression using the TCI in clinical samples [3, 8–11] and among students [12, 13]. 

A number of studies have shown that HA is positively correlated with depressive mood [2, 3, 10, 11, 14]. In particular, the HA subscales, HA1 (Anticipatory Worry) and HA4 (Fatigability), are more strongly correlated with mood than the other subscales [13, 15]. The relationship between depression and NS overall is inconsistent [8, 16], but there is clear evidence of a negative correlation between the subscale NS1 (Exploratory Excitability) and depressive symptoms [8, 11, 17]. Regarding RD, high levels of RD have been found to protect against the development of depression [11]. For SD, Peirson and Heuchert [13] found a significant negative correlation between SD subscales and mood, whereas Hansenne et al. [8] found no difference on SD scores between subjects with and without depression. Recently, one study found a negative correlation between SD and depression in a clinical sample after different treatments [3]. Most research has shown that low SD is associated with depression [2, 11, 18]. The C subscale Social Acceptance may be influenced by mood [8, 17]. 

It is also important to highlight that no studies valuate the relationship between temperament and depression in the general population during specific life situations, such as pregnancy. Although the presence of depression during and after pregnancy is currently considered a risk factor for the development of pathology in the child [19], few studies are available in the literature about the influence of temperamental characteristics of pregnant women on depression. Some data is available about the postpartum period, however. For example, Josefsson et al. [20] investigated whether women with postpartum depression differ in personality traits from healthy postpartum women. Results showed that HA and ST scales were higher, while SD and C were lower, in women with postpartum depression than the control group. 

This study is the first step of a longitudinal research aimed to identify predictors (temperament and mood) of family functioning/parenting style evaluated at 6 months, 1 year, and 3 years from child's birth. The aim of this first phase of our longitudinal study is to evaluate the relationship between depression and personality in men and women who are expecting a baby. In particular, we are interested in understanding how the pregnancy condition influences the mood of both the pregnant woman and her husband. 

## 2. Method

### 2.1. Participants

102 expectant parents participated to the study: 65 pregnant women and 37 husbands. Eighteen husbands of pregnant women did not participate at the prenatal courses from which the sample was recruited and ten husbands did not agree to participate to the study.

The mean age is 34.11 years for the women group (sd = 4.55) and 36.70 for the men group (sd = 4.99). Women were all primiparas and Caucasian; pregnant adolescents and single mothers were excluded from the sample. 

All socioeconomic classes were represented in the sample: all subjects reported their socioeconomic status according to seven categories from 1 to 7 to determine the educational level and the profession of subjects (SES) [21].

The characteristics of participants are summarized in Table 1.

### 2.2. Materials

The Temperament and Character Inventory—Revised Form (TCI-R) [7] is a self-report 5-point Likert scale, with 240 items. The questionnaire is used for the evaluation of personality according to the psychobiological model of Cloninger et al. [4, 5].

The Beck Depression Inventory (BDI) [22] is a 21-question multiple-choice self-report inventory for measuring the severity of depression in terms of its affective, physiological, and cognitive features.

### 2.3. Procedure

The sample recruited volunteers during prenatal courses in the Obstetrical Unit of an Italian Clinical Hospital. Subjects completed the TCI-R and BDI in the last quarter of pregnancy.

The questionnaires were administrated during one session of the prenatal course that was conducted by a psychologist. Subjects gave their written informed consent, although the questionnaires were anonymous.

### 2.4. Statistical Analysis

The two groups, pregnant women and their husbands, were compared on socio-demographic characteristics (age, education, occupation, and socioeconomic status).

In order to evaluate differences on depression level and temperament characteristics between women and men, Analysis of Variance (one way ANOVA) was performed between the two groups on Beck and TCI scores. 

Moreover, correlations (Pearson *r*) were calculated between age, Beck score, and all TCI scales, respectively on women and men samples. Subsequently, Hierarchical Multiple Regression Equations were computed, separately for pregnant women and their husbands, to reveal predictive relationships between temperament/character characteristics and depression level according to gender. Specifically, the Beck score was considered as the dependent variable, while age, TCI temperament scales (HA, RD, NS, and PS), and TCI character scales (SD, C, and ST) were included as predictors.

## 3. Results

ANOVA revealed significant differences between women and men on age (*F* = 7.15, *P* = .01), but there were no differences in SES (*F* = .29; *P* = .59). There were significant differences between men and women on education (Chi² = 6.86; *P* = .03) but not on occupation (Chi² = 7.43; *P* = .11).

Variance analysis also revealed that pregnant women had higher levels of depression (*F* = 4.16; *P* = .04), higher RD scores (*F* = 5.18; *P* = .03), and higher ST scores (*F* = 4.21; *P* = .04) compared to their husbands (see Table 2).

In the women, a moderate positive correlation was found between scores for depression and HA (*r* = .32; *P* = .01). In the men, in contrast, significant correlations were found between depression level and, respectively, age (*r* = .38; *P* = .02) and SD score (*r* = −.54; *P* = .00).

Hierarchical Multiple Regression Analysis in the pregnant women group showed that HA and SD were both significant predictors of the level of depression. Specifically, HA explained 12% of the variance and SD explained another 18%, while the inclusion of other variables did not increase the variance explained (see Table 3). Analysis in the husbands showed that only the SD score was a significant predictor of depression level, explaining 31% of variance (see Table 3).

## 4. Discussion

As expected from previous observations in the general population, we found that pregnant women report higher levels of depression prenatally than do their husbands. In fact, epidemiological data from around the world reveal that women are twice as likely as men to experience depression [23]. Due to the lack of a control group recruited from general population, we canot attribute the level of depression to the clinical status of the women. However, extensive literature supports the hypothesis that women are particularly vulnerable to mood disorders during crucial periods of the reproductive life cycle, particularly pregnancy [24]. 

Likewise as expected from the influence of maternal-child bonding on personality, we found that women have higher levels of RD than men. On average, compared to men, women are more sensitive to cues of social approval and to formation of emotional attachments [25]. We also found that pregnant women had higher scores on ST than their husbands. Even though Pelissolo and Lepine [26] find this gender difference also in adult general sample, it is possible that a woman who is carrying a new life inside may experience a state-specific outlook of unity and participation with all that exists and, consequently, she could be induced to feel more self-transcendent. We plan to evaluate changes in levels of personality traits longitudinally in these women so that we can evaluate changes in levels of personality scores with changes in pregnancy in the same woman. Finally, no differences are found between women and men on HA, even though previous data in the general population often find slightly higher scores in women than men. 

Regarding Multiple Regression Analysis, two dimensions of the TCI predict the level of depression in women, even after adjusting for age. Specifically, we found that pregnant women were more depressed if they were high in HA and low in SD. In contrast, HA did not significantly influence the level of depression in the husbands of the pregnant women. However, low SD was a strong predictor of depression in the husbands, explaining 31% of the variance. This extends earlier observations that low SD is a significant predictor of depression level in both men and women in the general population [13].

The results of our study should be considered in light of several limitations. First, the size of the sample is small, and a larger number of men may be needed to evaluate the weak effect of HA in men. Second, a control group made of notpregnant women would have allowed to attribute differences in personality, such as self-transcendence, to the state of pregnancy in the women. For example, our clinical experience suggests that the pregnant women are more exposed to social judgement and to strong external expectations to maintain a healthy state, such as a nonalcoholic and nutritious diet in order to promote healthy fetal and perinatal development. Consequently, pregnancy may be experienced as a stressful and demanding regime comparable to running an obstacle course or training for a marathon.

## 5. Conclusion

This study reports the first step of a longitudinal study designed to identify predictors (such as temperament, character, and mood) of family functioning and parenting style evaluated at 6 months, 1, and 3 years after a child's birth.

At this preliminary stage in the project, we have evaluated for the first time in the literature, at least to our knowledge, the relationship of depression with the temperament and character of parents who are expecting a child. 

Several of our findings are consistent with observations about people in the general population, but we have been able to extend them to a specific life situation (pregnancy). The finding that self-directedness is related to depression in both parents is particularly interesting because of its implications for improving subsequent child care. Maternal depression, in fact, can reduce mother-child bonding, increase shyness and insecure attachments in the child, impair personality development, and reduce the health and happiness of children throughout their life [19, 27]. Moreover, self-directedness is a relatively stable character trait with broad impact on adapting to life challenges like pregnancy and child care. Accordingly, it is imperative that we deepen our understanding of these preliminary findings with an increased sample size, a control group of nonpregnant women, and a long-term prospective followup with attention to the parenting roles of both the fathers and the mothers. Such observations will allow us to identify risk factors for the development of ill health and psychopathology in the children and to plan effective ways of intervening. Our data indicate that preventive interventions will require treatment of personality traits in the parents prior to or during prenatal psychoeducational courses.

## Figures and Tables

**Table 1:** Characteristics of participants.

Demographic data	Gender (F; M)	65; 37
	Age in years: Men group (M; SD)	36.70; 4.99
	Age in years: Women group (M; SD)	34.11; 4.55

Education women group	Primary school	1
	Secondary school	24
	University	40

Education men group	Primary school	5
	Secondary school	15
	University	17

Occupation: women group	Employee	40
	Self-employed worker	14
	Housewife	3
	Student	2
	Unemployed	6

Occupation: men group	Employee	22
	Self-employed worker	14
	Housewife	0
	Student	1
	Unemployed	0

SES Index	Women group (M; SD)	47.35; 14.58
	Men group (M; SD)	45.81; 12.70

**Table 2:** ANOVA between women and men groups on Beck and TCI scores.

	Women group		Men group		*F*	*P*
	m	Sd	m	sd		
Beck score	6.25	4.52	4.43	3.93	4.16	0.04
TCI-NS	100.92	17.76	100.35	17.63	0.03	0.88
TCI-HA	97.66	19.54	92.59	15.80	1.81	0.18
TCI-RD	104.62	16.87	96.86	15.94	5.17	0.02
TCI-P	115.83	24.87	111.57	26.35	0.66	0.42
TCI-SD	140.37	27.40	138.81	26.34	0.08	0.78
TCI-C	132.78	25.46	126.78	22.86	1.41	0.24
TCI-ST	75.38	19.25	67.89	14.69	4.21	0.04

**Table 3:** Percentage of variance, significance, B and *β* indexes, in Beck score explained by the different predictors (TCI temperament and character scales) in the Hierarchical Multiple Regression Equations (women and men samples).

	*R*² Change	B	*β*	*P*
Women group				
(1) Age	.00	−.03	−.03	n.s.
(2) NS	.01	−.01	−.04	n.s.
(3) HA	.12	.07	.32	.02
(4) RD	.01	−.07	−.28	n.s.
(5) P	.01	.02	.10	n.s.
(6) SD	.18	−.11	−.68	.04
(7) C	.05	.13	.71	n.s.
(8) ST	.00	.00	−.02	n.s.

Men group				
(1) Age	.15	.12	.15	n.s.
(2) NS	.00	.04	.16	n.s.
(3) HA	.09	.07	.29	n.s.
(4) RD	.03	.05	.19	n.s.
(5) P	.01	.04	.26	n.s.
(6) SD	.31	−.16	−1.10	.00
(7) C	.02	.04	.25	n.s.
(8) ST	.01	−.03	−.13	n.s.
